# Development of an *Escherichia coli*–*Lactobacillus casei* shuttle vector for heterologous protein expression in *Lactobacillus casei*

**DOI:** 10.1186/s40064-016-1760-1

**Published:** 2016-02-24

**Authors:** Namfon Suebwongsa, Viraphong Lulitanond, Baltasar Mayo, Panjamaporn Yotpanya, Marutpong Panya

**Affiliations:** Department of Microbiology and Research and Diagnostic Center for Emerging Infectious Diseases, Faculty of Medicine, Khon Kaen University, Khon Kaen, 40002 Thailand; Departamento de Microbiología y Bioquímica, Instituto de Productos Lácteos de Asturias (IPLA-CSIC), Paseo Río Linares, s/n, 33300 Villaviciosa, Asturias Spain; College of Medicine and Public Health, Ubon Ratchathani University, Ubon Ratchathani, 34190 Thailand

**Keywords:** *Lactobacillus casei*, Plasmid, Cloning vectors, Heterologous expression, Oral live vaccine

## Abstract

There is an increasing interest to develop various lactic acid bacteria (LAB) species as mucosal delivery vehicles, for which the development of a variety of cloning and expression systems for these bacteria is of primary importance. This study reports the complete nucleotide sequence of the cryptic plasmid pRCEID7.6 derived from the chicken probiotic LAB strain *Lactobacillus casei* TISTR1341. Sequence analysis and comparison showed that pRCEID7.6 is composed of nine putative open reading frames. The replicon origin of pRCEID7.6 consisted of untranslated origin of replication and translated replication protein B sequences. This region was used to construct *Escherichia coli*/*L. casei* shuttle vectors carrying erythromycin and chloramphenicol resistance genes as selective markers. Segregation and structural stability of the vectors in *L. casei* was sufficient for most genetic applications. The feasibility of this vector for heterologous protein expression in *L. casei* was determined by cloning in pRCEID-LC7.6, the gene encoding the nucleocapsid protein (NP), from the influenza A virus under the control of the homologous promoter from the lactate dehydrogenase gene. *L. casei* carrying this recombinant plasmid was shown to successfully express the NP protein. Therefore, this shuttle vector can be used for further study in the development of mucosal delivery vehicles.

## Background

*Lactobacillus casei* is a member of the lactic acid bacteria (LAB) that can be found in various environments, including that of animal and human intestines (Kandler and Weiss 1986). Besides generally recognized as safe (GRAS) status, many strains of LAB including a number of *L. casei* strains are also recognized as probiotics, defined as “live microorganisms that, when administered in adequate amounts, confer a health benefit on the host” (Hill et al. 2014). Some strains of *L. casei* have been demonstrated to exert probiotic properties, such as anti-infective, anti-tumor, and immunomodulating activity (Kim et al. 2006; Ohashi et al. 1989).

There is now a growing trend in the development of LAB as mucosal vaccines, as well as vehicles for the delivery of therapeutic and prophylactic molecules. A major advantage of using LAB as mucosal delivery vehicles is their ability to be engineered to express both homologous and heterologous proteins. Many studies have successfully used *L. casei* to express a variety of heterologous proteins of viral (Suebwongsa et al. 2013), bacterial (Liu et al. 2014), and mammalian origin (Qiu et al. 2013). One critical step to develop *L. casei* and other LAB as mucosal delivery vehicles is to construct the expression systems for these bacteria. Generally, most of the genetic manipulations in the development process are preferred to be done in *Escherichia coli* than in *Lactobacillus* species, thus *E.coli/Lactobacillus* shuttle vectors are the essential part of the process.

The construction of *E. coli/Lactobacillus* shuttle vectors is usually based on replicons, a DNA region that necessary for plasmid replication, derived from both *E. coli* and *Lactobacillus* species. The replicon of later species is derived from cryptic plasmid of *Lactobacillus* species. For the effective expression system for either homologous or heterologous proteins, the basic biological properties of plasmid vector, for example mode of replication, copy number, and stability, are key factors (Shareck et al. 2004). Previously, the researchers had determined the complete nucleotide sequence from three of the four plasmids derived from *L. casei* TISTR 1341, a probiotic strain isolated from chickens obtained from the Thailand Institute of Scientific and Technological Research (TISTR). The replicons from the largest and the smallest plasmids, pRCEID13.9 and pRCEID2.9 respectively, have already been successfully used to construct *E. coli/Lactobacillus* shuttle vectors (Panya et al. 2012).

In this study, the fourth plasmid of *L. casei* TISTR 1341, pRCEID7.6, was sequenced and its sequence analyzed. The replicon of this plasmid was then used to generate a new *E. coli/Lactobacillus* shuttle vector. The vector was successfully used for the expression of NP, a synthetic gene based on the nucleocapsid protein (NP) gene of the influenza A virus, in *L. casei*. Expression of this protein would allow its use as a mucosal delivery vehicle for oral immunization.

## Methods

### Bacterial strains and their growth conditions

Bacterial strains used in this study are listed in Table 1. *Lactobacillus* strains were cultured statically in de Man Rogosa Sharpe (MRS) medium (Difco, East Molesey, UK) at 37 °C. *E. coli* cells were grown in Luria–Bertani (LB) broth at 37 °C with vigorous shaking. When needed, antibiotics were added to the media as follows: for *E. coli*, ampicillin (100 µg/ml), chloramphenicol (10 µg/ml); for *L. casei*, erythromycin (2.5 µg/ml), chloramphenicol (5 µg/ml), and tetracycline (10 µg/ml). White/blue selection was done on LB agar supplemented with ampicillin, 20 mg/ml of 5-bromo-4chloro-3-indolyl-β-d-galatopyonoside (X-Gal) and 0.4 M of isopropyl-β-d-thiogalactopyranoside (IPTG) (Sigma-Aldrich, St. Louis, MO, USA).

**Table 1:** List of bacterial strains, plasmids, and oligonucleotide primers utilized in this study

Materials	Relevant properties	Source or reference
Bacteria		
*Escherichia coli* XL-1 Blue	White/blue screening	Stratagene, La Jolla, CA
*Lactobacillus* *casei* TISTR1341	Source of pRCEID7.6 native plasmid	TISTR
*Lactobacillus casei* RCEID02	Plasmid-free *L. casei* TISTR1341-derivative	Panya et al. (2012)
*Lactobacillus casei* ATCC393	Plasmid-free stain	ATCC
Plasmids		
pUC19E	Ap^r^, Em^r^, pUC19 carrying the Em^r^ gene from pE194 at the *Sma*I site	Leenhouts et al. (1991)
pGEM-T easy	Ap^r^, M13ori, T-overhang cloning vector	Promega, MD, USA
pRCEID-LC2.9	Ap^r^, Em^r^, *E. coli/L. casei* shuttle vector based on pRCEID2.9	Panya et al. (2012)
pRCEID-LC13.9Tc	Ap^r^, Tc^r^, pRCEID-LC13.9 derivative, the Tc^r^ gene was inserted at *Sac*I site	Panya et al. (2012)
pNZ8048	Cm^r^, *Nco*I site used for translational fusions, standard vector	Mierau and Kleerebezem (2005)
Oligonucleotides	Sequence (5′–3′)	
p6.8F-1	gggtcagttttgccttatg	This study
P6.8R-1	ctggcaatgactttgcgga	This study
p6.8F	attgcttggatcctctggcatgacaaac (*Bam*HI)	This study
p6.8R	ctttttggtatacggatccagtcgcta (*Kpn*I)	This study
SpeI-ldh_F	caaactagtagcttttagtcctcgtgaaaa (*Spe*I)	Suebwongsa et al. (2013)
pnisNdeI	attacatatgaagctcgcgttatcggtc (*Nde*I)	Suebwongsa et al. (2013)

Underlined nucleotides show introduced restriction enzyme sites, which are indicated in parenthesis

*TISTR* Thailand Institute of Scientific and Technological Research, *ATCC* American Type Culture Collection

### DNA isolation and purification

Plasmids from *E. coli* were isolated the by an alkaline lysis method, as described by Sambrook and Russell (2001). Plasmids from LAB were isolated by the O’Sullivan and Klaenhammer method (O’Sullivan and Klaenhammer O’Sullivan and Klaenhammer 1993). Total genomic DNA of *L. casei* was extracted and purified from the mid-log phase of bacterial cultures by using QIAamp DNA Mini Kit according to the instructions of the manufacturer (Qiagen, Hilden, Germany). Purified plasmids were verified by agarose gels electrophoresis, stained with ethidium bromide and visualized under UV light, and photographed.

### General DNA manipulation

General DNA manipulations were performed as described by (Sambrook and Russell 2001). Restriction endonuclease and T4 ligase were obtained from Fermentas (Fermentas, USA), *Taq* and *Pfu* polymerases were obtained from RBC (RBC, Taiwan). PCR amplicons and DNA fragments from agarose gels were purified using the Gel/PCR DNA Fragments Extraction Kit (RBC, Taiwan) as recommended by the manufacturer. Nucleotide sequences of the recombinant plasmids were verified by sequencing using a MegaBACE 1000 sequencer (BioDesign Co. Ltd., Pathumthani, Thailand).

### Plasmid sequencing and assembly

The contig2310 with a length of 6814 bp had been obtained as reported elsewhere (Panya et al. 2012). This sequence has been found to share high homology to several plasmids from LAB and proved to be unrelated to all other plasmid sequences from *L. casei* TISTR 1341. The researchers assumed the contig could be a major part of a plasmid with the length of 7.6 kb observed by agarose gel electrophoresis in *L. casei* TISTR 1341 (Panya et al. 2012). Thus, DNA from the band corresponding to that plasmid was extracted from a gel, purified, and used as a template to amplify the remaining sequence with conventional PCR using the primers p6.8F-1 and p6.8R-1 (Table 1). The amplicon obtained was cloned into pGEM-T easy vector and sequenced by using the primers M13F(−40) and M13R(−40) (Table 1). The obtained sequence was assembled with that of the previous contig2310 by using CLC Workbench 5.6 program.

### Analysis of the plasmid nucleotide sequence

Nucleotide sequence similarity searches were performed using the BLAST program at the NCBI database (http://www.ncbi.nlm.nih.gov/BLAST/). Open reading frames (ORFs), Direct (DR) and Inverse (IR) repeat sequences, restriction endonuclease sites, and plasmid maps were determined using Clone Manager 7.0 software. The putative promoter and ribosome binding site (RBS) sequences were searched for by comparing with the consensus sequences (TTGACA for −35 box, TATAAT for −10 box, and AGGAGG for RBS). Protein structure and motives were searched for by using the Pfam search tool (http://pfam.sanger.ac.uk/).

### Plasmid transfer

Plasmids were introduced into either *E. coli* or *L. casei* by electroporation. *E. coli* electroporation was performed using a Gene Pulser apparatus (Bio-Rad, Richmond, CA, USA) as described by Dower et al. (1988). Preparation of competent cells and electroporation of *L. casei* was performed as previously described by (Chassy and Flickinger 1987).

### Construction of *E. coli/L. casei* shuttle vector

The cloning procedure to obtain the pRCEID7.6-derived shuttle vector is shown in Fig. 2. Initially, a DNA sequence containing the *ori*, and *repB* genes from pRCEID7.6 was amplified by PCR using primers p6.8F and p6.8R (Table 1). The amplified segment was cloned into pGEM-T Easy vector resulting in a pGEM:pRCEID7.6Rep. The pRCEID7.6 replicon was recovered from pGEM:pRCEID7.6Rep by double *Aat*II/*Nde*I digestion and cloned into *Aat*II/*Nde*I-digested pUC19E, a pUC19 derivative contain *E. coli* replicon that does not replicate in *L. casei* but contains an erythromycin resistance gene as selective marker (Leenhouts et al. 1991), resulting in the construct pRCEID-LC7.6. To change the selective marker from erythromycin-resistant gene (Em^R^) to chloramphenicol-resistant gene (*cat*), the pRCEID-LC7.6 was digested with *Sal*I to remove the Em^R^ gene and was self-ligated, giving rise to pRCEID-LC7.6ery-. The chloramphenicol resistant gene (*cat*) was obtained from pNZ8048 by double *Sal*I/*Spe*I digestion and was inserted into *Sal*I/*Spe*I-digested pRCEID-LC7.6ery-, resulted in pRCEID-LC7.6Cm.

### Segregational and structural stability of the constructs

The segregational and structural stability of a pRCEID-LC7.6 shuttle vector was studied in *L. casei* RCEID02, a plasmid-free derivative from *L. casei* TISTR 1341. Transformants with pRCEID-LC7.6 were grown in MRS broth without erythromycin for approximately 200 generations. Every 20 generations, an aliquot of the culture was removed, diluted, and plated onto antibiotic-free MRS medium. One hundred colonies were selected and then replicated on MRS agar with and without erythromycin. For segregational stability, colonies grown on MRS agar with and without antibiotics were counted and calculated as follow: (N_A_/N_NA_) × 100, where N_A_ and N_NA_ is number of colonies appearing on MRS plate with and without erythromycin, respectively. For plasmid structural stability was checked by restriction analysis of plasmids isolated from colonies grown on MRS agar supplement with erythromycin at approximately 20, 40, 60, 80 and 100 generation intervals.

### Determination of plasmid copy number

Real-time quantitative PCR was used to determine the relative copy number of pRCEID-LC7.6 in *L. casei* RCEID02 as described by (Lee et al. 2006). The copy number of the constructs was calculated using the formula N_relative_ = (1 + *E*)^−ΔCT^, where *E* is amplification efficiency of the target (Em^R^) and reference (*greA*) genes, and −ΔCT is the difference between the threshold cycle number of the reference gene and that of the target. DNA amplification and detection was performed in a Fast Real-Time PCR equipment (Applied Biosystems, Foster City, CA, USA) using SYBR^®^ Green (Power SYBR^®^ Green PCR Master Mix, Applied Biosystems).

### Compatibility of the construct

The compatibility of pRCEID-LC7.6Cm with other vectors was assayed in *L. casei* RCEID02. Competent cells of *L. casei* harboring pRCEID-LC7.Cm were electrotransformed with pRCEID-LC2.9 and pRCEID-LC13.9Tc. Transformants were grown on MRS agar supplemented with appropriate antibiotics. The colonies on MRS agar were randomly selected for plasmid isolation. The plasmid profile was verified by agarose gel electrophoresis.

### Construction of pRCEID-LC7.6:LdhL:NP:TT

The DNA segment containing the promoter of the lactate dehydrogenase (LdhL) gene, the synthetic gene encoding the NP protein of influenza A virus and a transcription terminator (TT) obtained from pNZ8048, was amplified from pLC13.9:LdhL:NP:TT (Suebwongsa et al. 2013) using primers SpeI-ldh_F and pnisNdeI (Table 1). The amplicon was digested with *Spe*I/*Nde*I and inserted into pRCEID-LC7.6 digested with the same restriction endonuclease enzymes, resulting in pRCEID-LC7.6:LdhL:NP:TT.

### Detection of NP by Western blotting analysis

The bacterial cell lysates obtained by sonication from overnight cultures of *E. coli* and *L. casei* RCEID02 transformants containing pRCEID-LC7.6:LdhL:NP:TT were subjected to SDS-PAGE (12 % of SDS-PAGE, 100 V for 2 h) and their proteins transferred from the gel to a nitrocellulose membrane (Bio-Rad, Hercules, California, USA) by using the Trans-Blot^®^ SD Semi-Dry Electrophoretic Transfer Cell (Bio-Rad, Hercules, California, USA). The NP protein on the membrane was detected by a chemiluminescent detection system (CL-XPosure Film; Thermo Scientific, West Palm Beach, Florida, USA) using anti-Avian Influenza A Nucleoprotein antibody (Abcam, Cambrideg, UK), HRP-conjugated anti-rabbit IgG antibody (Abcam, Cambrideg, UK), and SuperSignal West Pico Chemiluminescent Substrate (Thermo Scientific, West Palm Beach, Florida, USA).

### Nucleotide sequence accession number

The complete nucleotide sequence of pRCEID7.6 was deposited in the GenBank database under accession number JN793951.

## Results

### Sequencing, sequence comparison and assemblage of pRCEID7.6

Initially, the contig2310 with a total length of 6814 bases showed a nucleotide identity of 96 and 87 % of the segments embraced by the positions 696–3639 and 5944–6814 respectively to sequences of plasmid pCD01 (AY662330.1) from *Lactobacillus paracasei* NFBC338, strongly suggesting that the contig may be part of pRCEID7.6, the missing plasmid of *L. casei* TISTR 1341 (Panya et al. 2012). To verify this possibility and to complete the nucleotide plasmid sequence, primers in opposite orientation were designed based on the sequence flanking both sides of the contig and used in PCR amplifications using as a template plasmid pRCEID7.6 isolated from a gel. A single amplicon of 1 kb was obtained which was subsequently cloned in *E. coli* in the pGEM-T vector and double stranded sequenced. Finally, assemblage of the new sequence to that of contig2310 produced a total 7604 bp which, after analysis, was considered to constitute the whole molecule of pRCEID 7.6.

### Sequence analysis of pRCEID7.6

**Fig. 1:**
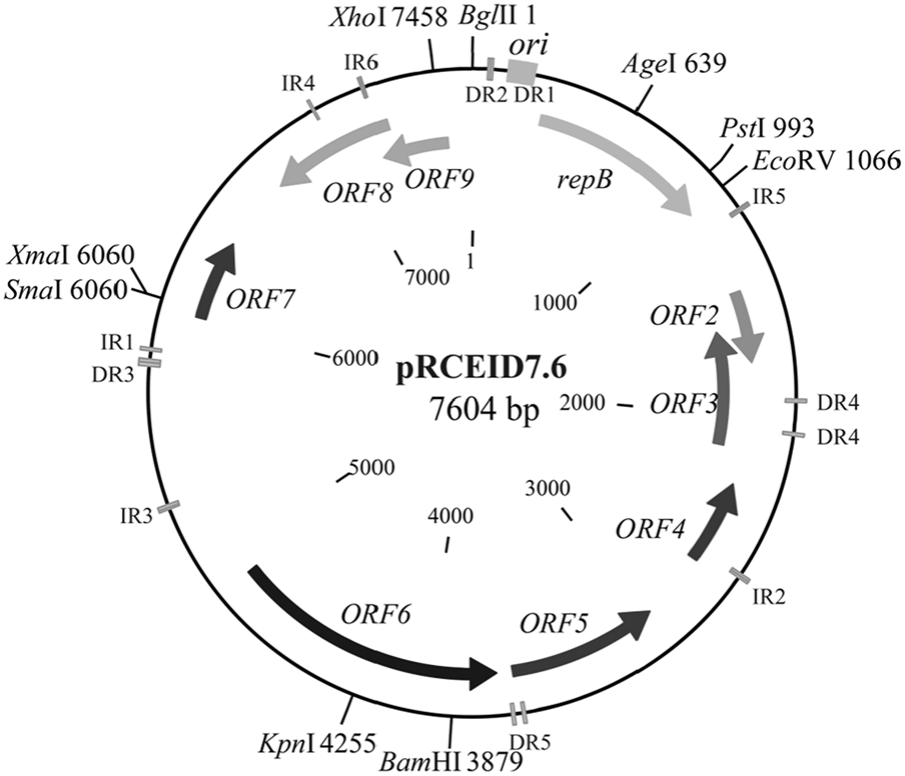
Physical and genetic map of the plasmid pRCEID7.6 (7604 bp) from *L. casei* TISTR1341. *Arrows* indicate the length and direction of the ORFs. Relevant unique restriction enzyme sites are indicated. The *ori* region of the plasmid is denoted by a *solid box*. *DR* direct repeats, *IR* inverted repeats

The genetic organization of the plasmid pRCEID7.6 is depicted in Fig. 1. Nucleotide and deduced amino acid comparisons against the GenBank database predicted that pRCEID7.6 was composed of nine ORFs larger than 50 amino acids. The products of these ORFs and the most homologous protein in databases to each of them are listed in Table 2.

**Table 2:** Open reading frames (ORFs) identified in the 7604-bp plasmid pRCEID7.6 from *L. casei* TISTR1341

Gene/ORF	% GC content	5′ end position	3′ end position	No. of aa.	Known protein with the highest homology (microorganism)	% amino acid identity (length)	GenBank accession no.
*repB*	39	297	1076	259	Plasmid replication protein, RepB	100 (259)	YP_003329273.1
					Plasmid pCD01p07 (*L. paracasei* subsp. *paracasei*)		
ORF2	42	1461	1772	103	Hypothetical protein of plasmid pCD01p08 (*L. paracasei* subsp. *paracasei*)	97 (100)	YP_003329274.1
ORF3^a^	41	1609	2148	179	Putative replication protein of plasmid pCD01 (*L. paracasei* subsp. *paracasei*)	100 (179)	YP_003329275.1
ORF4^a^	36	2316	2684	122	Hypothetical protein LSEI_A19 of plasmid1 (*L. casei* ATCC334)	100 (122)	YP_796451.1
ORF5^a^	39	2977	3627	216	Hypothetical protein of plasmid1 (*L. casei*)	100 (216)	YP_005351865
ORF6^a^	38	3696	4886	396	C-terminus part of type IC specificity subunit protein (*L. rhamnosus*)	92 (180)	ZP_04439481.1
ORF7	43	6027	6377	116	Plasmid replication protein of pLR001 (*L. rhamnosus* HN001)	99 (115)	YP_002221586.1
ORF8^a^	45	6701	7237	178	Transposase (*L. paracasei* subsp. *paracasei* 8700:2)	96 (165)	ZP_04672204.1
ORF9^a^	45	7168	7488	106	Transposase tnp4 (*L. casei* BL23)	100 (106)	YP_002221607.1
					Transposase (*L. rhamnosus* HN001)	100 (106)	YP_002221607.1
					Transposase (*L. buchneri* ATCC 11577)	100 (106)	ZP_03943910.1

^a^ORFs are encoded on the complementary strand

The product of three ORFs, *repB*, ORF3, and ORF7, encoded putative proteins involved in plasmid replication. The *repB* gene encoded a protein of 259 amino acids with 100 % amino acid identity with the replication protein B (RepB) of plasmid pCD01 (YP_003329273.1) from *L. paracasei*. Upstream of the *repB* gene of pRCEID7.6, a putative origin of replication (*ori*) region was observed, including a DR of 10-bp (ATACTTCTAA) repeated two times, and a tandem DR of 22-bp (ATAGGTCACCAAAAAGCACACG) repeated four times followed by a truncated repeat (ATAGGTCACCAAAAAG). This organization resembles the typical feature of the theta-replicating plasmid family of pUCL287 (Kanatani et al. 1995). Downstream of the 22-bp DR, a putative promoter (TTGtgtn15TtaAAT) was found. In addition, a possible ribosome binding site (RBS) (GAGGTG) downstream of the promoter was observed 7 bp upstream from the start codon of *repB* (ATG). Therefore, based on sequence similarity, pRCEID7.6 could be reasonably ascribed to the theta-mode of replication.

Surprisingly, the gene product of ORF3 and ORF7 also showed homology to replication-associated proteins. ORF3 encoded 179 amino acid that have a 100 % identity to whole replication protein of plasmid pCD01 (YP_003329275.1) from *L. paracasei* NFBC338. Similarly, the gene product of ORF7, a protein of 116 amino acids, showed 99 % (115 amino acid identity) sequence identity to a 116 amino acids of putative replication protein of plasmid pLR001 (YP_002221586.1) from *L. paracasei* HN001. However, since the immediate upstream sequence of these two ORFs lack the typical features of *ori* region, such as those described for *repB*, they are considered to be non-functional for the replication of pRCEID7.6

ORF2 and ORF4 encoded protein with unknown function. ORF2 encoded 103 amino acid with 97 % similarity to hypothetical protein of plasmid pCD01p08 (YP_003329274.1) from *L. paracasei*. ORF4 encoded a protein of 122 amino acids with 100 % similarity to an hypothetical protein of plasmid1 (YP_796451.1) from *L. casei* ATCC334. A conserved RBS (GGGAGA) was found at 7 bp upstream of the start codon (ATG) of ORF4. The gene product of ORF5 consisted of 216 amino acids with 100 % homology to an hypothetical protein of plasmid1 of *L. casei* ATCC334 (YP_796451). ORF8 and ORF9 were found to encode proteins responsible, most likely, for plasmid recombination and integration. ORF8 encoded a protein of 178 amino acid s with 96 % identity to transposases (ZP_04672204.1) from *L. paracasei* 8700:2. This protein is known as a member of transposase family with DDE_Tnp domain (PF01609). Similarly, ORF9 encoded a protein of 106 amino acids with 100 % identity to transposase (tnp4) (CAQ65752.1) from *L. casei* BL23 and transposase AEA57601.1 from *L. casei* BD-II.

ORF6 encoded a peptide very similar to the partial part of C-terminal domain of a type IC specificity subunit protein with Methylase_S domain (pfam, PF01420). This domain is also known as a member of target recognition domain (TRD) involved in bacterial restriction-modification system.

### Construction and characterization of the *E. coli/L. casei* shuttle vectors

**Fig. 2:**
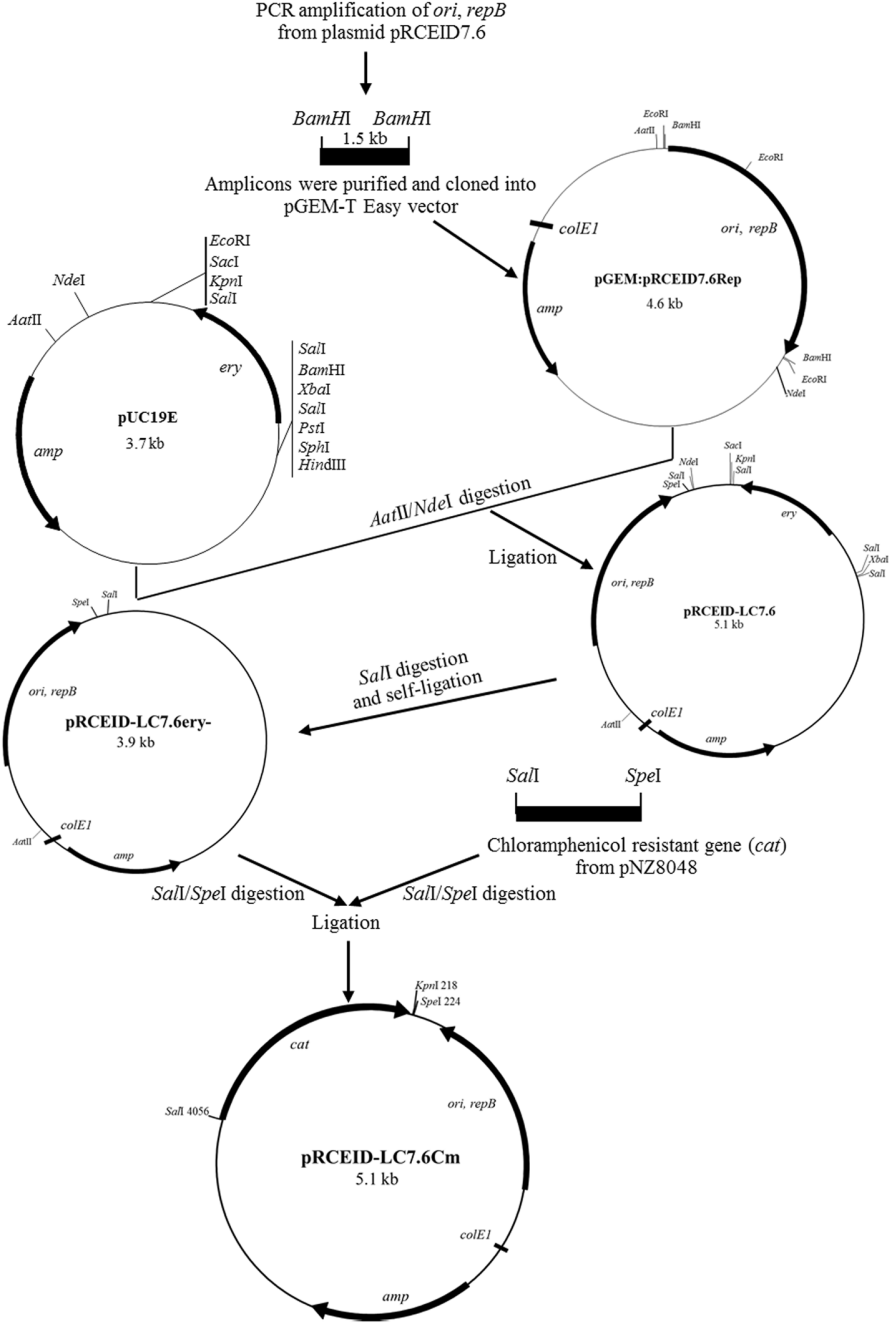
Construction of the shuttle vectors, pRCEID-LC7.6 and its derivatives, pRCEID-LC7.6ery-, and pRCEID-LC7.6Cm. *amp*, *ery* and *cm* = ampicillin, erythromycin, and chloramphenicol resistant genes respectively

Figure 2 shows the cloning steps for constructing the pRCEID7.6-derived *E. coli/L. casei* shuttle vectors. From the sequence analysis, it was considered that the pRCEID7.6 replication region (including the *ori* sequence and *repB*) could consist in the DNA segment encompassing the nucleotide positions from 135 to 1076. Primers were designed to amplify this region and the resulting amplicon was cloned in *E. coli* in pUC19E. As this vector carries an erythromycin resistance gene active in Gram-positives, the construct pRCEIC-LC7.6 was then electrotransformed into *L. casei* RCEID02 where it proved to drive plasmid replication.

**Fig. 3:**
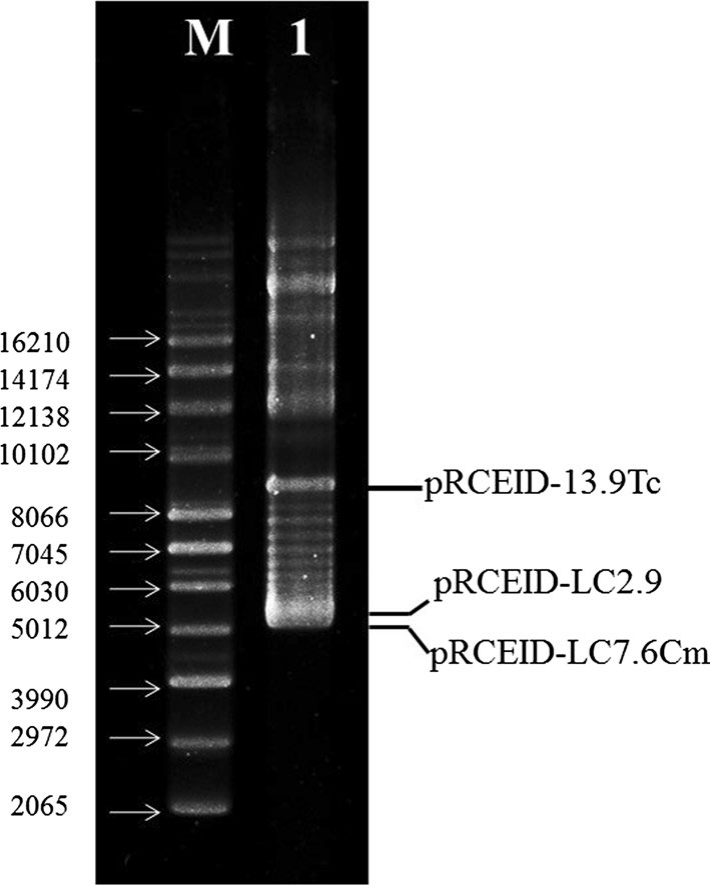
Ethidium bromide staining agarose gel of plasmid profile of *L. casei* RCEID02 harboring pRCEID-LC7.6Cm (5.1 kb), pRCEID-LC2.9 (5.3 kb), and pRCEID-LC13.9Tc (7.1 kb). *Lane 1* plasmid profile of *L. casei* RCEID02 harboring pRCEID-LC7.6Cm, pRCEID-LC2.9, and pRCEID-LC13.9Tc. *Lane M* Super coiled DNA ladder (Invitrogen, USA). On the *left*, sizes in base pairs of the different molecules of the ladder. The electrophoresis reaction was carried out by using 0.7 % agarose gel electrophoresis in ×1 TAE buffer at 50 voltages for 3 h

For the vector to be compatible with the two previous vectors developed from other plasmids from the *L. casei* TISTR 1341 strain, the erythromycin resistance gene of construct pRCEID-LC7.6 was changed by a chloramphenicol resistance gene, giving rise to plasmid pRCEID-LC7.6Cm (Fig. 2). It was found that pRCEID-LC7.6 could compatibly replicate with both pRCEID-LC2.9 and pRCEID-LC13.9Tc in the same bacterial host (Fig. 3).

**Fig. 4:**
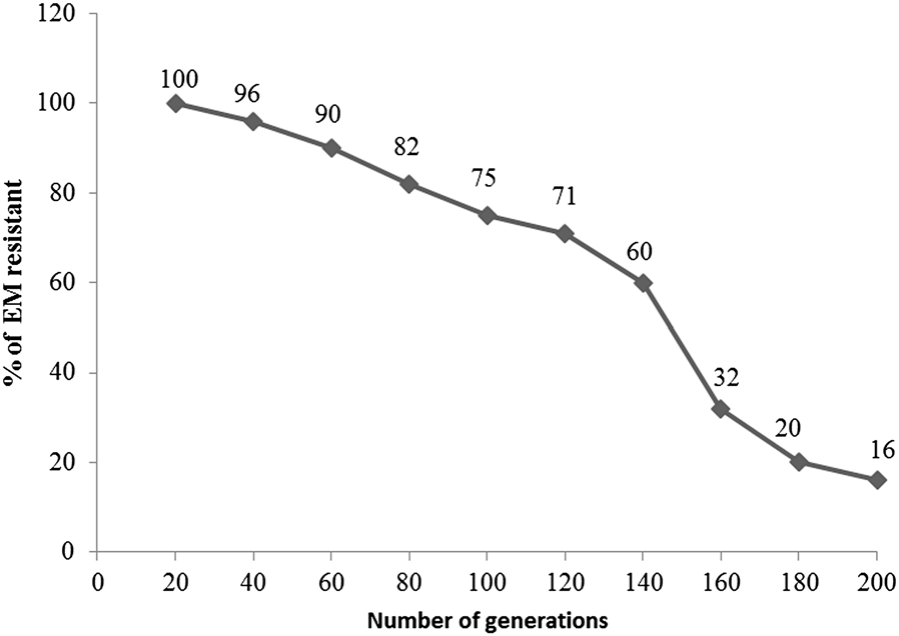
Segregational stability of the shuttle vectors pRCEID-LC7.6 in *L. casei* RCEID02. *L. casei* RCEID02 harboring these plasmids was culture in the absence of selective pressure, plated on the same condition, and assayed for plasmid maintenance by replica-plating onto erythromycin-containing MRS medium at approximately 20, 40, 60, 80, 100, 120, 140, 160, 180 and 200 generation intervals. *Em* erythromycin

**Fig. 5:**
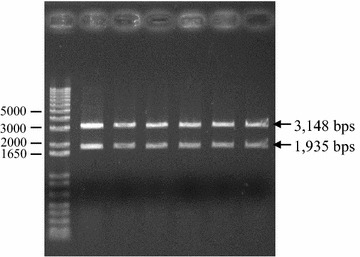
Ethidium bromide staining gel of *Hind*III-digested shuttle vector pRCEID-LC7.6. *Lane 2*–*6* *Hind*III-digested pRCEID-LC7.6 derived from the generation time of 20, 40, 60, 80 and 100, respectively. *Lane 1* the original vector pRCEID-LC7.9 digested with *Hind*III. *Lane M* MassRuler™ Express Reverse DNA ladder

Structural and segregational analysis in *L. casei* RCEID02 indicated that the vector pRCEID-LC7.6 was maintained in 75 and 16 % of the cells after 100 and 200 generations, respectively, in the absence of selective pressure (without antibiotics) (Fig. 4). Additionally, structural changes of plasmid after 100 generations were never observed (Fig. 5). The relative copy number per chromosome equivalent of the constructs was found to be 12 copies per chromosome in *L. casei* RCEID02.

### Expression of a heterologous protein in *L. casei* RCEID02 using pRCEID-LC7.6

**Fig. 6:**
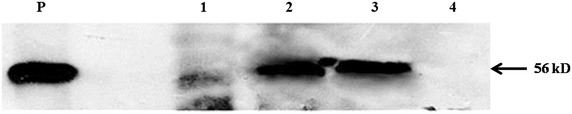
Western blot analysis of expressed NP in recombinant *L. casei* RCEID02 and *E. coli* XL-1 blue containing NP gene using goat anti-NP polyclonal antibody for detection. *Lane P* Influenza virus H1N1 infected cell lysate. *Lane 1* *E. coli* XL-1blue containing pRCEID-LC7.6. *Lane 2* recombinant *E. coli* XL-1blue containing pRCEID-LC7.6:LdhL:NP:TT. *Lane 3* recombinant *L. csei* RCEID02 containing pRCEID-7.6:LdhL:NP:TT. *Lane 4* *L. casei* RCEID02 containing pRCEID-LC7.6

**Fig. 7:**
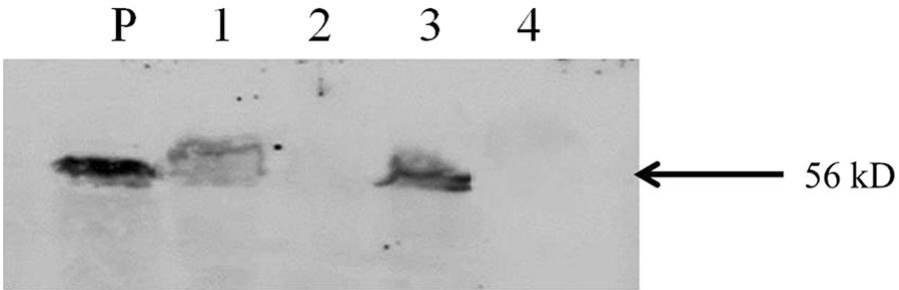
Western blot analysis of expressed NP in recombinant *L. casei* RCEID02 harboring different vectors using goat anti-NP polyclonal antibody for detection. *Lane P* Influenza virus H1N1 infected cell lysate. *Lane 1* *L. casei* containing pRCEID-7.6:LdhL:NP:TT. *Lane 2* *L. casei* containing empty pRCEID-7.6. *Lane 3* *L. casei* containing pRCEID-13.9:LdhL:NP:TT. *Lane 4* *L. casei* containing empty pRCEID-13.9

To determine whether pRCEID-LC 7.6 can be used for expression of heterologous proteins, the construct pRCEID-LC7.6:LdhL:NP:TT was developed. It is a pRCEID-LC7.6 derivative carrying an expression cassette composed of the *ldh* gene promoter, a codon-optimized, synthetic gene encoding the NP of influence A virus, and a transcription terminator derived by PCR from pLC13.9:LdhL:NP:TT (Suebwongsa et al. 2013) was introduced into *E. coli* XL-1 blue and *L. casei* RCEID02. Expression of the NP protein in both hosts was determined by Western blot analysis. As shown in the Fig. 6, a protein of the expected size of NP (56 kD), and identical to that of a cell lysate infected with the influenza virus H1N1 used as a control, was clearly revealed in transformants of the two species. In addition, the expression level of NP in *L. casei* RCEID02 after using the different plasmids, pRCEID-LC 7.6 and pRCEID-LC13.9 as backbone vector, was compared. It was found that that expression level of NP in *L. casei* RCEID02 was not difference as shown in Fig. 7.

## Discussion

*Lactobacillus casei* is a member of LAB that has drawn a lot of attention as the candidate for the development of mucosal delivery vehicles (Wen et al. 2012). This bacterial species has been recognized as safe and certain strains possess probiotic properties conferring various health benefits to the hosts (Dong et al. 2013). For example, *L. casei* strain Shirota was demonstrated to enhance NK cell activity by dairy intake (Takeda and Okumura 2007; Takeda et al. 2006). It has been demonstrated that oral administration of *L. casei* Shirota in humans can prevent and reduce the risk of the occurrence of bladder carcinoma (Aso et al. 1995; Ohashi et al. 2002). Besides probiotic properties, *L. casei* has the most essential property for development as a mucosal delivery vehicle, the ability to be genetically engineered. For genetic engineering in *L. casei* and other LAB, plasmid replicons are generally required to develop the genetic tools for manipulation. The researchers’ previous study (Panya et al. 2012) sequenced and analyzed three cryptic plasmids present in *L. casei* TISTR1341 and selected two replicons, one from pRCEID13.9 and the other from pRCEID2.9, to construct *E.coli/Lactobacillus* shuttle vectors, pRCEID-LC13.9 and pRCEID-LC2.9 respectively. Due to the high structural and segregational stability of pRCEID-LC13.9, this vector was successfully used for expressing the nucleocapsid protein of influenza A virus in *L. casei* RCEID02 (the plasmid-free derivative of *L. casei* TISTR1341) in the researchers’ subsequent study (Suebwongsa et al. 2013). In this present work, the pRCEID7.6 were completely sequenced and analyzed. The replicon of pRCEID7.6 was identified and selected to construct a new shuttle vector using pUC19E as backbone. The new vector, pRCEID-LC7.6, showed excellent structural stability as there was no structural change after 100 generations. For segregation stability, though the stability was less than that of pRCEID13.9 and pRCEID-LC2.9, and it was sufficient (75 and 16 % after 100 and 200 generations, respectively) for protein expression and other genetic processes in this system. Though not thoroughly verified, replication in other strain of *L. casei* is expected as pRCEID-LC13.9 and 2.9 were shown to be able to replicate in different *L. casei* strains (Panya et al. 2012). In addition, as this vector can replicate with both pRCEID13.9 and pRCEID2.9 in a single cell, this allows the development of more robust, naturally compatible vectors. Finally, pRCEID-LC7.6 was used for the expression of NP of influenza A virus under the control of lactate dehydrogenase promoter in *L. casei* RCEID02. The successful expression of a heterologous protein in *L. casei* in this study allows further promising development of this vector as a mucosal delivery vehicle in the near future.

## Conclusion

The present study reported the completion of the plasmid complement of *L. casei* TISTR 1341, which consists in four plasmids, pCREID2.9, pRCEID3.2, pCREID7.6, and pRCEID13.9. As the replicons of all four plasmids are naturally compatible, the development vectors based on these plasmid replicons would provide high flexibility for cloning and expression of homologous and heterologous proteins in *L. casei* and other LAB species. The suitability of the new cloning vector pRCEID-LC7.6 was demonstrated by cloning and expressing the nucleocapsid protein-encoding gene from the influenza A virus. Expression of this protein would allow its use as a mucosal delivery vehicle for oral immunization.
